# The Emigration of Young Women

**Published:** 1911-08-26

**Authors:** 


### THE EMIGRATION OF YOUNG WOMEN.

There is a Society, of which Princess Christian is President, to organise the emigration of young women to our Colonies. A brilliant woman writer has poured scorn upon the scheme as a form of husband hunting—a sport for which she has, quite comprehensibly, the greatest contempt. But Miss Cicely Hamilton ignores certain important aspects, both human and national, of the aims of the Society. The desire for a mate is an absolutely natural one. The mating instinct is, and ought to be, present in healthy men and women, and it is well to recognise the fact and give it its honourable place among the laws of nature. It is quite a different thing from the desire to be kept by someone else—a form of laziness which Miss Hamilton rightly despises. But, as a matter of fact, the life of the average wife, in all classes, is far from being one of luxurious idleness, and any extra honour that is paid to the matron beyond what is given to the maid is due rather to her extra responsibilities than to superior prowess in the chase for a mate. In our day, if never before, equal honour is paid to the woman who serves her country in other ways as is bestowed on the mother, whose main service is the continuing of the race. Indeed, the danger is that the obvious national services of the spinster may win more than their due meed of recognition and the commonplace business of maternity less. That marriage in itself has lost its old kudos is a good thing, since it tends to prevent women who would hate the responsibilities of married life from entering upon it; it is better that they should be selfish as spinsters than as wives.

But to admit this is a very different thing from saying that a girl should not hope for a husband as a man hopes one day to have a wife. The desire is on each side equally natural, right, and honourable. But many men of our nation go abroad, and we are thus left with a number of women—not superfluous in any harsh sense—but women for whom there are not husbands enough. And the men who have gone abroad marry, when they come to marry, the girls whom they find at hand, who are not always of the same blood as themselves. "Propinquity, propinquity, propinquity," as Miss Edgeworth long ago pointed out, is the greatest of all match-makers. And, in spite of what the law says, the child is the mother's. Quite rightly do we speak of the mother-tongue. The language, the ideas, learned in infancy, abide with us all our lives, and it is from the mother that these are derived, and not the father. If therefore we wish British ideas to obtain throughout the British Empire, we must try to provide British mothers for the future imperial race. And this can be attained only by the judicious emigration of British women.

To let all our emigrating men marry wives of other races, without any compensating intermarriage on the other side, would be to develop unnecessary differences of thought and aim in every different part of the Empire. If Greater Britain is to hold together, the women of Britain must do their part as well as the men, and the way in which they can do their part best is to go to the Colonies where men have preceded them. Of course, it is obvious that not every English girl is suited to be a colonist. Life is not an easy thing for the settler's wife, and only girls who possess (besides the primary essential of good health) energy, initiative, and adaptability, are really suitable. But such girls are not lacking in any class—and the others are sure to come home in disgust! A training in the duties which are to be expected in the new sphere to which the girl emigrant proposes to go is eminently desirable. That will, in the first place, weed out a good many girls who think of Colonial life as a perpetual hunting or shooting house-party, and experience must do the rest. But it is desirable, in the interests of the Empire, that young women should be encouraged to emigrate.

